# Hyperglycemia-Associated Dysregulation of O-GlcNAcylation and HIF1A Reduces Anticancer Action of Metformin in Ovarian Cancer Cells (SKOV-3)

**DOI:** 10.3390/ijms19092750

**Published:** 2018-09-13

**Authors:** Aneta Rogalska, Ewa Forma, Magdalena Bryś, Agnieszka Śliwińska, Agnieszka Marczak

**Affiliations:** 1 Department of Medical Biophysics, Institute of Biophysics, Faculty of Biology and Environmental Protection, University of Lodz, Pomorska 141/143, 90-236 Łódź, Poland; aneta.rogalska@biol.uni.lodz.pl (A.R.); agnieszka.marczak@biol.uni.lodz.pl (A.M.); 2 Department of Cytobiochemistry, Institute of Biochemistry, Faculty of Biology and Environmental Protection, University of Łódź, Pomorska 141/143, 90-236 Łódź, Poland; ewa.forma@biol.uni.lodz.pl (E.F.); magadalena.brys@biol.uni.lodz.pl (M.B.); 3 Department of Nucleic Acids Biochemistry, Faculty of Medicine, Medical University of Lodz, Pomorska 251, 92-213 Łódź, Poland

**Keywords:** metformin, hyperglycemia, ovarian cancer, apoptosis, O-GlcNAcylation, HIF1A gene

## Abstract

Although cancer cells need more glucose than normal cells to maintain energy demand, chronic hyperglycemia induces metabolic alteration that may dysregulate signaling pathways, including the O-GlcNAcylation and HIF1A (Hypoxia-inducible factor 1-alpha) pathways. Metformin was demonstrated to evoke metabolic stress and induce cancer cell death. The aim of this study was to determine the cytotoxic efficiency of metformin on SKOV-3 cells cultured in hyperglycemia and normoglycemia. To identify the potential mechanism, we assessed the expression of O-linked β-*N*-acetlyglucosamine transferase (OGT) and glycoside hydrolase O-GlcNAcase (OGA), as well as hypoxia-inducible factor 1-alpha (HIF1A) and glucose transporters (GLUT1, GLUT3). SKOV-3 cells were cultured in normoglycaemia (NG, 5 mM) and hyperglycemia (HG, 25 mM) with and without 10 mM metformin for 24, 48, and 72 h. The proliferation rate, apoptotic and necrotic SKOV-3 cell death were evaluated. Real-Time qPCR was employed to determine mRNA expression of OGT, OGA, GLUT1, GLUT3, and HIF1A. Metformin significantly reduced the proliferation of SKOV-3 cells under normal glucose conditions. Whereas, the efficacy of metformin to induce SKOV-3 cell death was reduced in hyperglycemia. Both hyperglycemia and metformin induced changes in the expression of genes involved in the O-GlcNAcylation status and HIF1A pathway. The obtained results suggest that dysregulation of O-GlcNAcylation, and the related HIF1A pathway, via hyperglycemia, is responsible for the decreased cytotoxic efficiency of metformin in human ovarian cancer cells.

## 1. Introduction

Ovarian cancer is one of the leading cause of death among women. Recently, it was demonstrated that the risk of ovarian cancer is increased among diabetic patients [1]. Diabetes-related pathologies such as insulin resistance, chronic inflammation and high free ovarian steroid hormones are thought to be responsible for this coexistence [2]. Moreover, numerous clinical studies identified that hypoglycemic medications modulate the risk of cancer development. Metformin, the drug of the first choice in type 2 diabetes mellitus treatment, was found to protect from cancer development. Furthermore, experimental studies have revealed that metformin exhibited antiproliferative activity against various cancer cells. The drug was also reported to inhibit the growth of ovarian cancer cells [2,3]. It is widely believed that AMPK (5′AMP-activated protein kinase) activation and inhibition of mTORC (mammalian target of rapamycin complex) are mechanisms of anticancer action of metformin. The drug was also demonstrated to reduce tumorigenesis through metabolic alteration as a result of the mitochondrial respiratory complex I inhibition [3,4]. 

The typical hallmark of cancer cells is metabolic reprogramming [5]. Cancer cells, to satisfy their energy demand as well as carbon and nitrogen indulgence for macromolecules synthesis, consume an increased amount of glucose and glutamine. This results in the alteration of metabolic and signaling pathways, including inhibition of LKB1 (liver kinase B1)/AMPK and activation of mTORC pathways [6]. Activated mTORC promotes the expression of critical cell growth and metabolic regulators, including hypoxia-inducible factor 1-alpha (HIF1A). This transcription factor favors the expression of numerous genes that contribute to increased consumption of glucose and glutamine [7]. One of the downstream effectors of HIF1A is glucose transporters (GLUT), which are responsible for glucose uptake. In cancer cells, both HIF1A and GLUT1 were found to be upregulated [7,8].

Approximately 3–5% of the total glucose entering a cell is converted into UDP-N-acetylglucosamine (O-GlcNAc), in the hexosamine pathway. O-GlcNAc is used as a substrate for posttranslational modifications of intracellular proteins and thereby regulates their activity. O-GlcNAcylation controls protein function directly or by competing with phosphorylation sites. In response to altered nutrients and metabolic stress, O-GlcNAcylation was found to regulate cellular signaling and transcription regulatory pathways, including the HIF1A pathway [9,10]. Two enzymes regulate the level of O-GlcNAcylation; O-linked β-*N*-acetlyglucosamine transferase (OGT) and glycoside hydrolase O-GlcNAcase (OGA). An elevated level of O-GlcNAcylated proteins was found in different cancers [11]. 

Cell metabolism is closely connected with cell death pathways through the mitochondria, which play a crucial role in both metabolism and apoptosis. Metformin was found to affect cellular metabolism by targeting mitochondrial respiratory complex I and exerts direct anticancer action through the induction of cancer cell death or inhibition of cancer cell growth. It is well known that cancer cells are hypersensitive to metabolic stress, such as glucose or glutamine deprivation, and will undergo apoptosis if nutrients are limited [12]. However, little is known about how glucose excess influences the pro-apoptotic action of metformin in tumor cells. To answer this question, we aimed to determine the antiproliferative efficacy of metformin in human ovarian cancer (SKOV-3) cells cultured in normoglycemia (NG) and hyperglycemia (HG). As metformin evokes metabolic alteration, we assessed the expression of OGT and OGA, as well as HIF1A, GLUT1 and GLUT3. 

## 2. Results

### 2.1. Antiproliferative Efficacy of Metformin in SKOV-3 Cells Depends on the Level of Glycemia

**Figure 1:**
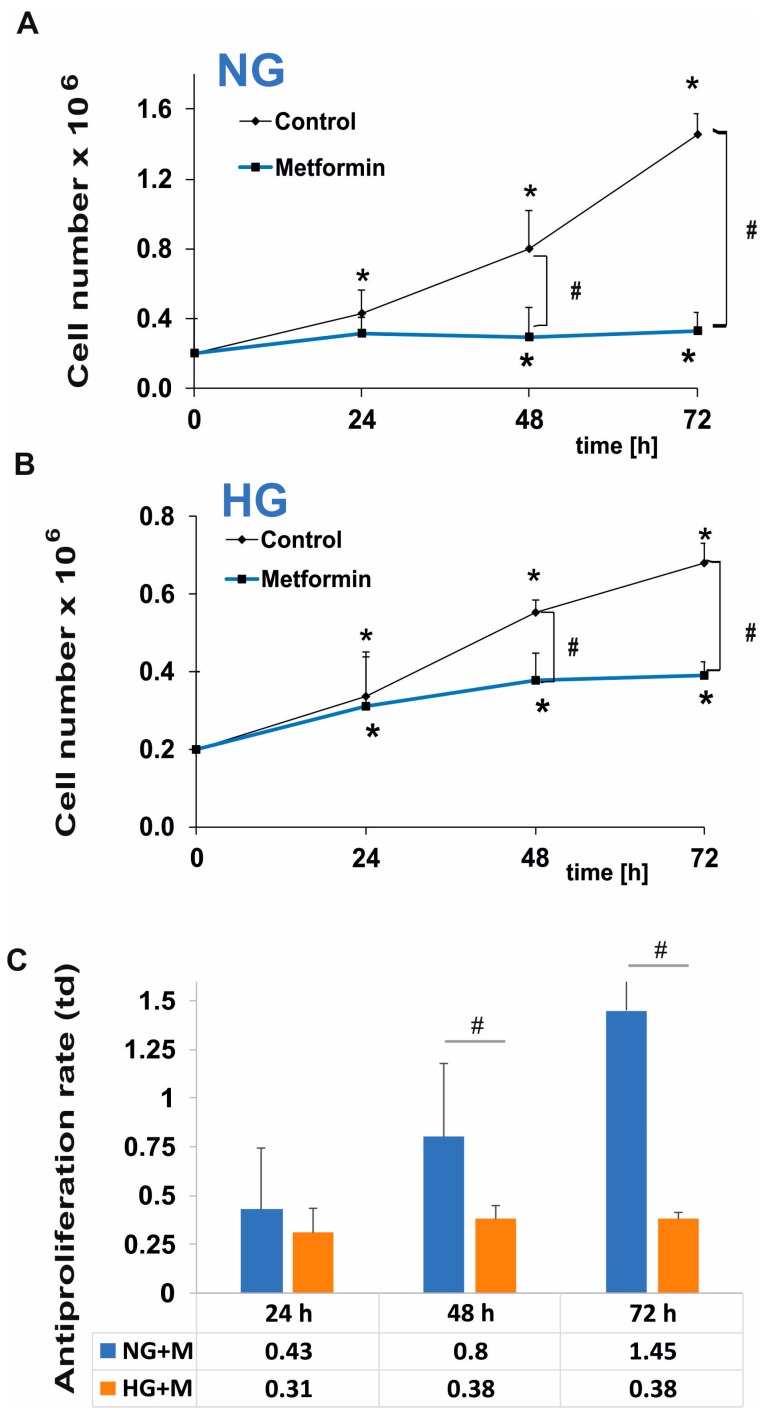
Antiproliferative action of metformin in SKOV-3 cells is reduced by hyperglycemia. SKOV-3 cells were cultured in normoglycemia (**A**) and hyperglycemia (**B**) and simultaneously treated with 10 mM metformin for 24, 48, 72 h. The cell number was determined using the trypan blue method and the proliferation rates were calculated (**C**). NG + M—cells cultured in normoglycemia and treated with metformin, HG + M—cells cultured in hyperglycemia and treated with metformin, td—proliferation rate. Results are presented as means ± SD of four independent experiments. (*) Statistically significant differences in comparison to untreated cells at time point “0”, *p* < 0.05; (#) statistically significant differences between the cells exposed to metformin in comparison to unexposed cells at the same time point, *p* < 0.05.

The antiproliferative efficacy of metformin in NG is presented in Figure 1A. According to the method described by Rogalska [13], we determined the values of doubling time of SKOV-3 cells cultured in NG (28 h) and HG (41 h). As displayed in Figure 1A, exposure to 10 mM metformin in NG induced a considerable time-dependent growth inhibition. Figure 1B shows the proliferation of SKOV-3 cells cultured in HG in the presence of metformin. As indicated by the value of doubling time, SKOV-3 cells cultured in HG needed more time to increase their number two times. This indicates that exposure to HG caused a decrease in proliferation efficacy of SKOV-3 cells. The growth rate of SKOV-3 cells cultured in HG in the presence of 10 mM metformin was also low. Furthermore, by comparing proliferation rates (td) in NG and HG, we revealed that HG significantly reduced the antiproliferative efficacy of metformin in SKOV-3 cells, as presented in Figure 1C. 

### 2.2. Both HG and Metformin Evoked Morphological Changes of SKOV-3 Cells

**Figure 2:**
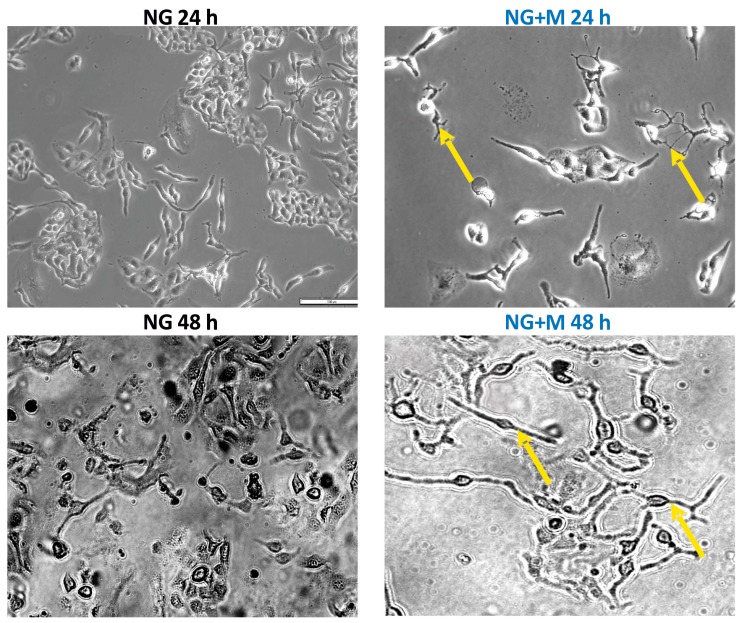

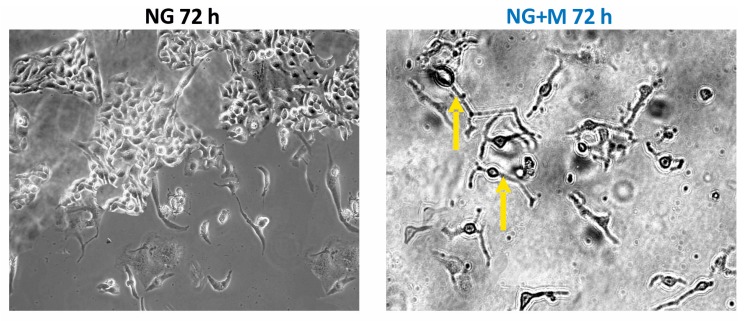
The morphology of SKOV-3 cells treated for 24–72 h with metformin (10 mM) in normal glucose medium examined under an inverted microscope (Olympus IX70, Japan), (scale bar = 100 μm), elongated, thin cells (yellow arrows).

**Figure 3:**
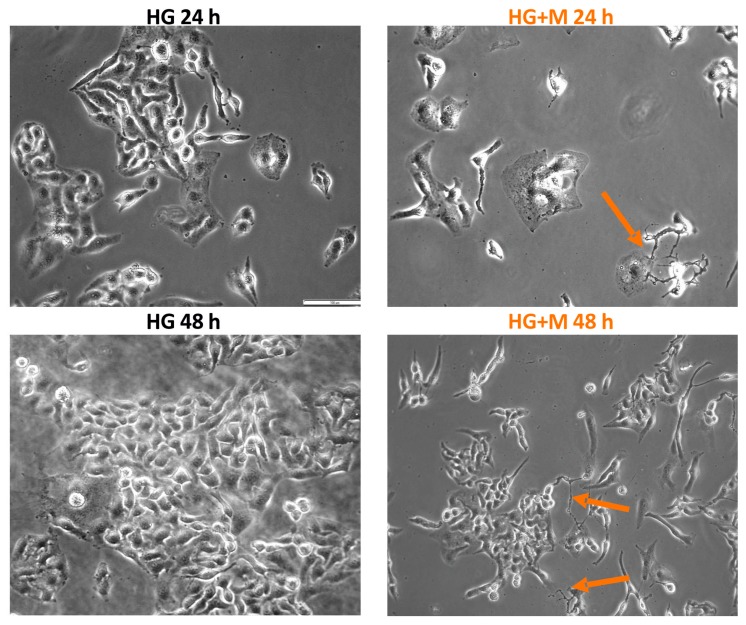

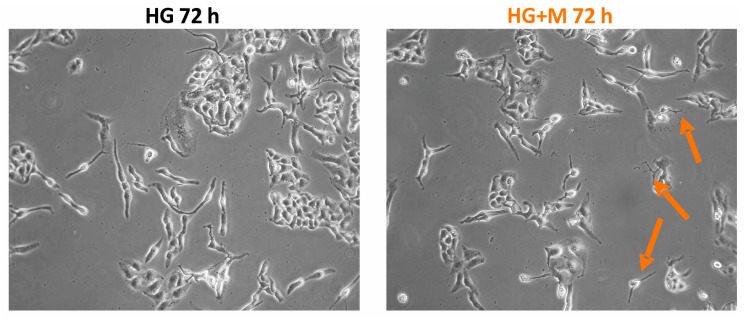
The morphology of SKOV-3 cells treated for 24–72 h with metformin (10 mM) in high glucose medium examined under an inverted microscope (Olympus IX70, Japan), (scale bar = 100 μm), elongated, thin cells (orange arrows).

The morphology of SKOV-3 cells cultured in NG and HG treated with 10 mM metformin for 24, 48, and 72 h examined under an inverted optical microscope (Olympus IX70, Tokyo, Japan) [14] is presented in Figure 2 and Figure 3. A small percentage of SKOV-3 cells cultured in NG for 24, 48, and 72 h exhibited morphological changes such as elongation and thinning. In turn, exposure of SKOV-3 cells cultured in NG to metformin pronouncedly increased the number of deteriorated cells in a time-dependent manner. Elongated, single thin cells were detected after just 24 h of exposure to metformin and their number increased after prolonged treatment with the drug (Figure 2). In the case of SKOV-3 cells cultured in HG, morphological changes appeared after 48 h of incubation. Both culture in HG for 48 h and 72 h caused elongation and thinning of the cells, while we also detected slightly smaller cells after 72 h. We found distinctly small, elongated and disintegrated SKOV-3 cells cultured in HG and metformin. Even 24 h exposure to metformin induced deterioration of cells cultured in HG. We also noted that prolonged treatment with metformin (48, 72 h) led to cell disintegration and detachment of the cells from the culture well surface (Figure 3).

### 2.3. Metformin Induced Mainly Apoptosis in NG, and Both Apoptosis and Necrosis in HG 

**Figure 4:**
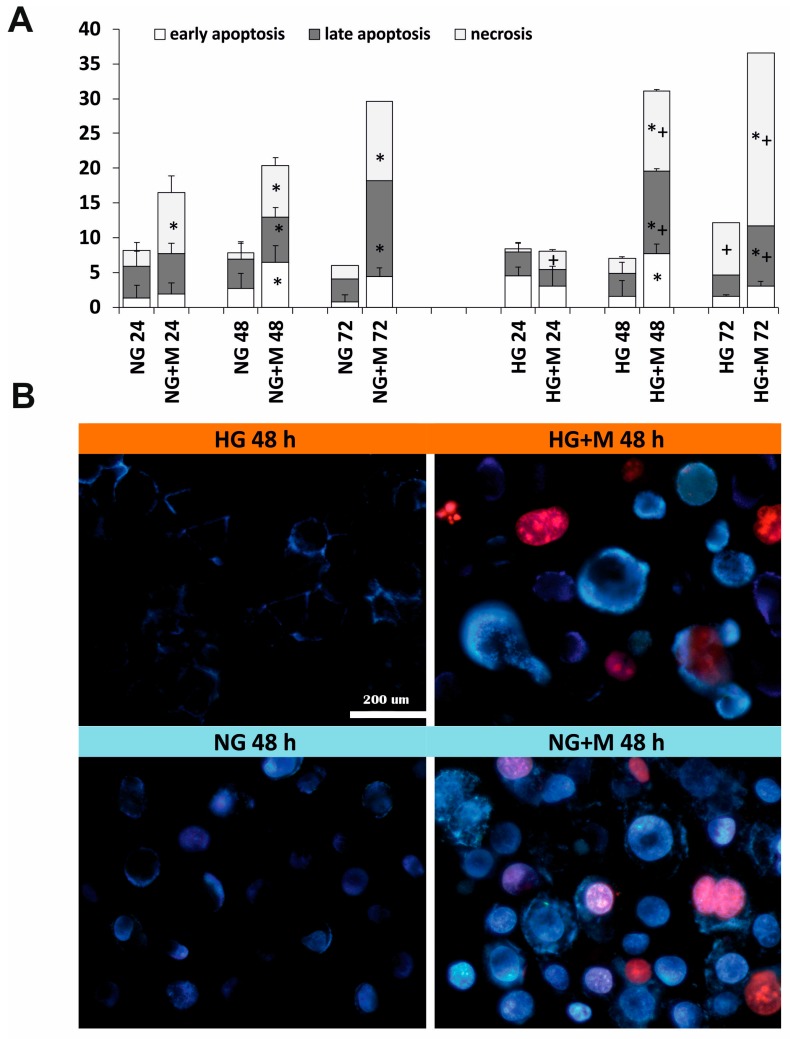
Metformin induced mainly apoptosis in NG and both apoptosis and necrosis in HG. (**A**) The percentage of early apoptotic, late apoptotic and necrotic cells detected at 24, 48, and 72 h of the culture of SKOV-3 cells in NG and HG in the presence and absence of 10 mM metformin. Results are presented as means ± S.D. of four experiments. NG—cells cultured in normoglycemia, NG + M—cells cultured in normoglycemia and treated with metformin, HG—cells cultured in hyperglycemia, HG + M—cells cultured in hyperglycemia and treated with metformin. (*) Statistically significant differences between the cells exposed to metformin in comparison to unexposed cells at the same time points, *p* < 0.05; (+) statistically significant differences between metformin treated cells in different time points, *p* < 0.05. (**B**) The morphological changes of SKOV-3 cells, which were cultured in NG and HG with and without exposure to 10 mM metformin for 48 h and stained with Hoechst 33258 and PI, visualized by fluorescence microscopy (Olympus IX70, Japan; bar 200 µm).

Figure 4 depicts the typical early apoptotic, late apoptotic, and necrotic morphological changes of SKOV-3 cells cultured in NG and HG in the presence or absence of 10 mM metformin. To discriminate between apoptotic or necrotic SKOV-3 cell death induced by metformin in NG and HG, double staining with Hoechst 33258 and PI with subsequent microscopic analysis was performed. These fluorescent dyes emit several types of fluorescence and differ in their ability to penetrate cells. Blue-fluorescent Hoechst 33258 goes through the intact membrane of live cells and allows for the observation of apoptosis-related chromatin structure changes. It stains the condensed chromatin of apoptotic cells brighter than the looser chromatin of normal cells. In turn, viable and early apoptotic cells with intact cell membranes exclude the red-fluorescent PI. Thus, only late apoptotic and necrotic cells, with the loss of membrane integrity, take up PI. The following morphological changes are detected by the double staining with Hoechst 33258 and PI: Chromatin condensation, cell shrinkage and nuclear fragmentation, apoptotic bodies, plasma membrane and cell disintegration.

A quantitative analysis of the fractions of early apoptotic, late apoptotic, and necrotic cells, is exhibited in Figure 4A. We found that both SKOV-3 cells cultured in NG and HG did not differ significantly in the percentage of early apoptotic, late apoptotic and necrotic cells. However, a visible increase in the percentage of necrotic cells was noted for SKOV-3 cells culture in HG for 72 h. We found that metformin induced, in a time-dependent manner, both apoptosis and necrosis in SKOV-3 cells cultured in NG. A significant increase in the number of apoptotic and necrotic cells was observed in HG-cultured SKOV-3 cells exposed to metformin for 48 and 72 h. In the case of culture in NG, early and late apoptosis was the major pathway of SKOV-3 cell death caused by metformin. Whereas, a higher percentage of early and late apoptotic, compared to necrotic cells was detected for SKOV-3 cells cultured in HG and exposed to metformin for 48 h. In turn, after 72 h of exposure to metformin, we found a pronouncedly high percentage of necrotic cells in HG-cultured SKOV-3 cells.

The typical early apoptotic, late apoptotic and necrotic morphological changes induced by metformin in SKOV-3 cells cultured in NG and HG for 48 h are presented in Figure 4B. The small percentage of dead SKOV-3 cells cultured in NG for 48 h represented apoptotic cells. It seems that there was an increased percentage of necrotic SKOV-3 cells cultured in HG. To conclude, mainly apoptosis, rather than necrosis, was involved in the metformin-induced death of SKOV-3 cells cultured both in NG and HG. 

### 2.4. Both Metformin and HG Affect Expression of OGT and OGA in SKOV-3 Cells

**Figure 5:**
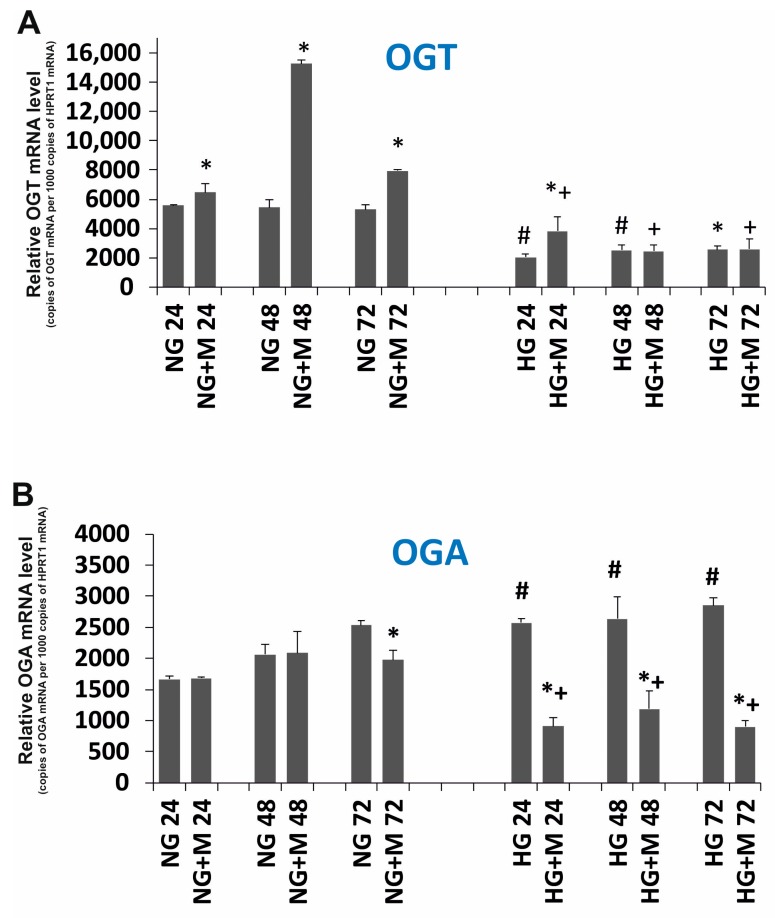
Relative (**A**) OGT (O-linked β-*N*-acetlyglucosamine transferase); (**B**) OGA (O-GlcNAcase) mRNA expression detected after 24, 48, and 72 h in metformin-treated and untreated ovarian cancer cell line SKOV-3 cultured in NG and in HG. NG—cells cultured in normoglycemia, NG + M—cells cultured in normoglycemia and treated with metformin, HG—cells cultured in hyperglycemia, HG + M—cells cultured in hyperglycemia and treated with metformin, (*) Statistically significant differences between the cells exposed to metformin in comparison to unexposed cells at the same time points, *p* < 0.05; (#) statistically significant differences between cells cultured in HG in comparison to those cultured in NG at the same time-points *p* < 0.05; (+) statistically significant differences between metformin-treated cells in HG in comparison to metformin-treated cells cultured in NG at the same time points, *p* < 0.05.

To explore the role of O-GlcNAcylation in SKOV-3 cell death induced by 10 mM metformin upon NG and HG, we evaluated the mRNA expression levels of OGT and OGA. The relative OGT mRNA and OGA mRNA levels are shown in Figure 5. We observed that culture in HG diminished the OGT mRNA level at each time point studied as compared to culture in NG. In NG-cultured SKOV-3 cells, exposed to 10 mM metformin, the level of OGT mRNA at 48 h and 72 h was markedly elevated. Whereas, the level of OGT mRNA seemed to be unaffected by 10 mM metformin in HG-cultured SKOV-3 cells. The obtained results suggest that HG influenced the level of OGT mRNA more than metformin. 

We found that HG did not change significantly the OGA mRNA level in SKOV-3 cells in comparison to NG. Metformin also did not affect the level of OGA mRNA in SKOV-3 cells cultured in NG. However, a noticeable decrease in the OGA mRNA level evoked by metformin was observed in HG at all time points studied.

### 2.5. Expression Changes of GLUT1, GLUT3 and HIF1A Evoked by HG and Metformin

**Figure 6:**
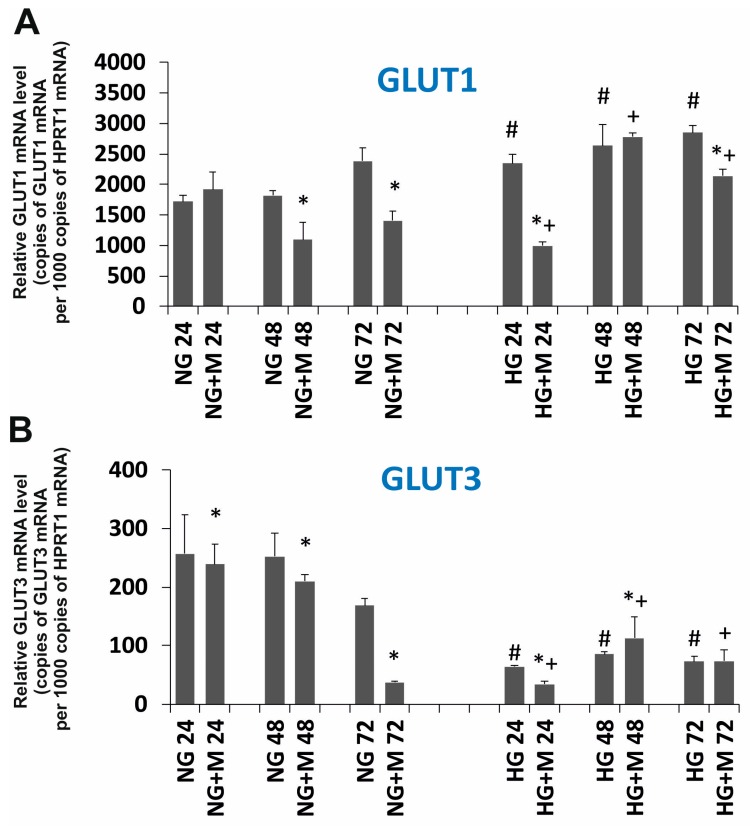

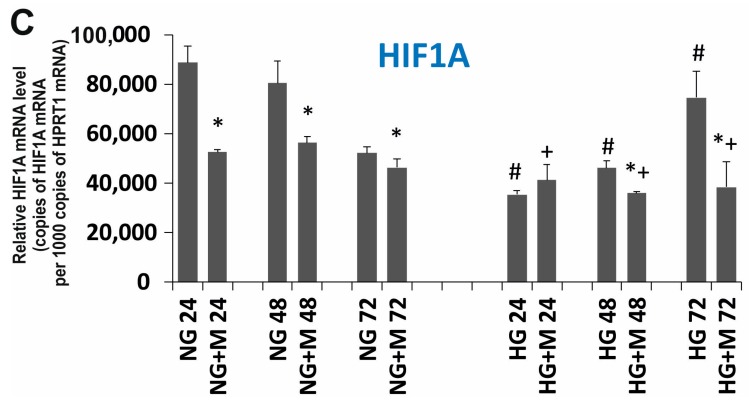
Relative (**A**) SLC2A1 (GLUT1); (**B**) SLC2A3 (GLUT3); (**C**) HIF1A mRNA expression detected after 24, 48, and 72 h in a metformin-treated and untreated ovarian cancer cell line (SKOV-3) cultured in NG and in HG. NG—cells cultured in normoglycemia, NG + M—cells cultured in normoglycemia and treated with metformin, HG—cells cultured in hyperglycemia, HG + M—cells cultured in hyperglycemia and treated with metformin. (*) Statistically significant differences between the cells exposed to metformin in comparison to unexposed cells at the same time points, *p* < 0.05; (#) statistically significant differences between cells cultured in HG in comparison to those cultured in NG at the same time points *p* < 0.05; (+) statistically significant differences between metformin-treated cells in HG in comparison to metformin-treated cells cultured in NG at the same time points, *p* < 0.05.

The relative GLUT1, GLUT3 and HIF1A mRNA levels are shown in Figure 6A–C. As presented in Figure 6A,B, it seems that the expression of GLUT1 is significantly higher than the expression of GLUT3 in SKOV-3 cells. This observation may suggest that GLUT1 plays a dominant role in glucose transport in SKOV-3 cells. The relative expression of GLUT1 did not change significantly in HG compared to NG. However, exposure to metformin for 48 and 72 h caused a distinct decreased expression of GLUT1 in SKOV-3 cell cultured in NG. SKOV-3 cells cultured in HG treated with metformin presented a decreased expression of GLUT1 after 24 h and unchanged expression at the rest of the time points. The relative GLUT3 expression was diminished in SKOV-3 cells cultured in HG compared to NG (Figure 6B). Metformin evoked a significant decrease in the level of GLUT3 mRNA in SKOV-3 cells cultured in NG for 48 and 72 h. It seems that the drug did not influence the expression of GLUT3 in HG-cultured cells.

We observed that the culture of SKOV-3 cells in HG affected the expression of HIF1A in comparison to the culture in NG. We found that the level of HIF1A mRNA diminished in SKOV-3 cells in NG, especially at 72 h. On the contrary, in SKOV-3 cells cultured in HG for 72 h, the HIF1A level was elevated (Figure 6C). Interestingly, both in the case of SKOV-3 cells cultured in NG and HG, metformin reduced the expression of HIF1A, in the normoglycemic condition after 24 and 48 h of exposure to metformin and in the hyperglycemic condition after 48 and 72 h of treatment with metformin.

## 3. Discussion

According to the American Cancer Society, ovarian cancer ranks fifth in cancer deaths among women, accounting for more deaths than any other cancer of the female reproductive system. This cancer mainly develops among older women, especially after menopause. The incidence of type 2 diabetes grows with age, therefore, the coexistence of ovarian cancer and diabetes also increases with age. The major symptom of diabetes—chronic hyperglycemia—may significantly influence the effectiveness of ovarian cancer chemo- and radiotherapy. Metformin, the first line hypoglycemic agent in the therapy of Type 2 diabetes mellitus (T2DM), was demonstrated to inhibit the growth of various cancers. Therefore, we aimed to evaluate the anticancer efficacy of metformin toward human ovarian cancer cells cultured in normoglycemia and hyperglycemia. Since glucose exposure causes metabolic alteration that affects O-GlcNAcylation and HIF1A pathway, we examined the expression of OGT, OGA, HIF1A, GLUT1, and GLUT3.

Interestingly, we found that the proliferation of SKOV-3 cells cultured in hyperglycemia was significantly lower in comparison to normoglycemia. This is unusual for cancer cells because it is widely accepted that high glucose stimulates proliferation by satisfying energy and carbon needs [15]. Since O-GlcNAcylation is sensitive to metabolic state and is involved in tumor growth in vitro [16], we determined the expression of OGT and OGA mRNA. We found that HG caused a significant decrease in the expression of OGT and a slight increase in OGA in SKOV-3 cells. Thus, our results suggest that lower proliferation of SKOV-3 cells exposed to HG may be associated with a decrease in O-GlcNAcylation. It is contrary to the general view that by reducing O-GlcNAcylation (via a decrease in OGT) tumor growth is inhibited [10].

We demonstrated that cytotoxic action of metformin on SKOV-3 cells was significantly reduced in HG in comparison to NG. This result is in agreement with findings reported by others, who found a decreased anticancer efficacy of metformin in breast cancer cells cultured in HG (MCF7, MDAMB231 and SKBR3) [17]. In addition, the reduced efficacy of other chemotherapeutics was also reported for liver cancer cells (HepG2, Bel-7402) cultured in HG [16]. Ikemura noted that in diabetic mice, the growth of adenocarcinoma in the colon was slower and that chemotherapy with oxaliplatin and fluorouracil was less effective [18]. According to Zhuang, high glucose provides fuel for glycolytic metabolism that maintains ATP levels in the cell despite inhibition of mitochondrial oxidative metabolism by metformin. As glucose is consumed and AMPK is not effectively activated by metformin, cancer cells do not have enough fuel to maintain glycolytic metabolism. As a result, ATP drops in the cell, leading to energy collapse and cell death [19]. In turn, Karnevi provided molecular evidence that the decreased anticancer action of metformin in pancreatic cancer cells exposed to HG involved the insulin/insulin-like growth factor-1 (IGF1) pathway. Exposure to high glucose levels promoted IGF-1-mediated PKB (protein kinase B, Akt) activation, which correlated with stimulated AMPKSer^485^ phosphorylation and impaired AMPKThr^172^ phosphorylation, resulting in reduced anti-proliferative and apoptotic effects by metformin [20].

The next step of our research was to identify the potential mechanism responsible for different types of metformin’s cytotoxicity toward SKOV-3 cells in NG and HG. As O-GlcNAcylation is sensitive to metabolic alteration, we examined the expression of O-GlcNAcylation regulators. We found a significant increase in the level of OGT mRNA in SKOV-3 cells cultured in NG and exposed to metformin. This observation may be a result of AMPK activation by metformin. Evidence indicated that AMPK directly phosphorylates OGT and increases its level [21,22]. Interestingly, our results revealed that the expression of OGT in cells exposed to both HG and metformin or only exposed to HG was pronouncedly diminished. These observations may suggest that HG changes O-GlcNAcylation status in SKOV-3 cells more than metformin. The O-GlcNAcylation status strictly depends on the balance between the level of OGT and OGA. The effect of metformin alone and HG alone on the expression of OGA was small. However, Pagesy detected a significantly elevated OGA level in diabetic patients [23]. In turn, simultaneous exposure to HG and metformin of SKOV-3 cells was associated with a marked decreased expression of OGA in comparison to SKOV-3 cultured only in NG or cultured in NG and metformin. These results may imply that metabolic stress caused by HG and metformin affects OGA level and thereby may play a significant role in the O-GlcNAcylation status in SKOV-3 cells. 

A cancer cell presents metabolic reprogramming, which is manifested by alterations in numerous signaling pathways. One of them is the HIF1A pathway that is launched by the hyperactive mTOR pathway in different cancer cells, including ovarian cancer [6,7,24]. The HIF1A pathway was also found to be elevated as a result of increased O-GlcNAcylation (via increased OGT) in response to metabolic alteration [9,10]. HIF1A mediates the adaptation of cells to low oxygen, mainly through upregulation of its effectors participating in glycolytic metabolism with the glucose transporter family (GLUT) at the head. The influence of glucose concentration on HIF1A expression was reported to be dependent on the cell type [25]. In our study, we demonstrated that SKOV-3 cells cultured in HG displayed lower expression of HIF1A and GLUT3 and an unchanged expression of GLUT1 in comparison to cells cultured in NG. The obtained results indicate that the effect of metformin on the HIF1A pathway was dependent on glycemic condition. In SKOV-3 cells cultured in NG we observed that metformin decreased the expression of HIF1A, GLUT1 and GLUT3. This is consistent with the result found by Alves in Sertoli cells, which revealed metformin enhanced glycolytic flux [26]. Wang found that by reducing HIF1A, the level of GLUT1 also decreased in human glioblastoma cells [27]. In turn, it was demonstrated, that metformin inhibited HIF1A and suppressed the expression of glucose transporters (GLUT1, GLUT3) and regulatory enzymes of the glycolytic pathway in cervical tumor cells [28]. Oppositely, in SKOV-3 cells cultured in HG, metformin slightly diminished HIF1A expression and did not influence GLUT1 and GLUT3 expression. Consistently, Qi demonstrated that in oral squamous cell carcinoma cells metformin significantly reduced the expression of HIF1A [29]. In agreement with our data, Ece reported a significantly decreased level of HIF1A in patients with type 2 diabetes and breast cancer receiving metformin [30]. It is believed that metformin specifically reduces HIF1A expression as a result of the inhibition of ATP synthesis and this action is independent of AMPK activation [31]. Interestingly, Xiao indicated that HG, via the generation of reactive oxygen species, is responsible for the reduced expression and activation of HIF1A [25]. It is clearly seen that the influence of metformin on the expression of GLUT1 and GLUT3 in SKOV-3 cells cultured in HG is negligible. Taken together, these observations suggest that, despite decreasing HIF1A expression, metformin may not run glycolytic flux in SKOV-3 cells cultured in HG.

Multiple clinical trials indicate that metformin reduces the risk of cancer development in diabetic patients [32]. Furthermore, metformin was found to inhibit the progression of various cancers, including ovarian cancer. Kumar showed in a case-control study that patients with ovarian cancer treated with metformin had a significantly better 5-year survival (disease recurrences and cancer-specific mortalities) in comparison to patients with ovarian cancer not taking metformin [33]. However, little is known about metformin action in patients suffering from ovarian cancer without diabetes. Currently, a phase II clinical trial is ongoing to assess whether the addition of metformin to standard chemotherapy improves survival in non-diabetic ovarian cancer patients (NCT02122185; clinicaltrials.gov). The results of our current in vitro study revealed that metformin inhibited proliferation and promoted apoptosis of SKOV-3 cells more efficiently in normoglycemia than in hyperglycemia. Our findings are in line with previously reported evidence showing that low glucose intensifies metformin cytotoxicity toward breast and thyroid cancer cells [34,35]. The mechanism by which the glucose level influences metformin action is being investigated. Zhuang suggested that low glucose in the medium triggered metformin mediated ATP depletion and cell death by reducing metformin-stimulated glycolysis [19]. Furthermore, recently, Tang demonstrated that metformin induced apoptosis and inhibition of ovarian cancer cell growth was associated with a stronger AMPK activation by metformin in a low glucose medium, in comparison to a high glucose medium. They showed that metformin action involved epigenetic alteration since the treatment with the drug significantly reduced histone H3 lysine 27 trimethylation and polycomb repressor complex 2 (PRC2) levels [36]. All together, these findings support the need for the evaluation of metformin efficacy for the treatment of patients with ovarian cancer without diabetes.

**Figure 7:**
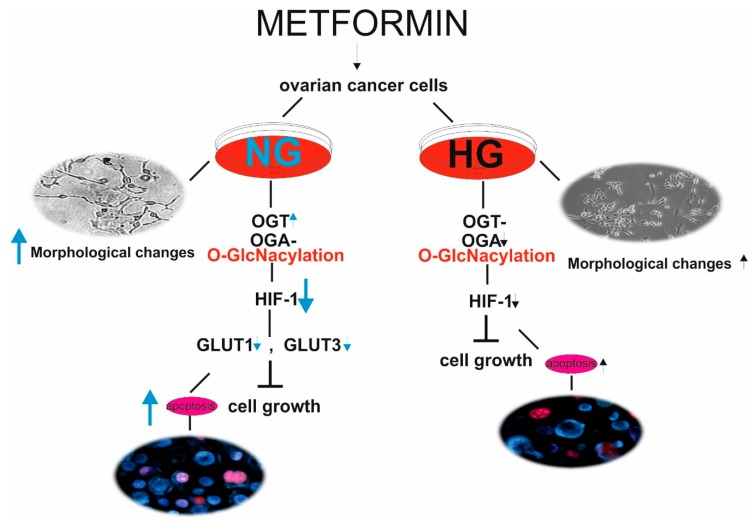
Metformin acts through a HIF1A/GLUT-dependent mechanism in SKOV-3 cells. The response of ovarian cancer cells to metformin depends on the level of glycemia, which affects diversely OGT and OGA expression and O-GlcNAcylation status. In NG, metformin increases the expression of OGT and downregulates HIF1A and GLUT expression leading to SKOV-3 cell apoptosis. HG dysregulates OGT and OGA expression and related HIF1A and GLUT expression preventing metformin-stimulated inhibition of growth and proliferation of SKOV-3 cells. Black arrows (HG conditions), blue arrows (NG conditions)—the height of arrow indicates intensification and direction of apoptotic changes, morphological changes and expression of the examined genes.

To conclude, our results indicate that metformin exhibits direct antitumor action on ovarian cancer SKOV-3 cells. The anticancer efficacy of metformin is higher when ovarian cancer cells were cultured in NG in comparison to culture in HG. To the best of our knowledge, this is the first study presenting that the response of SKOV-3 cells to metformin depends on the level of glycemia, which affects diversely OGT and OGA expression and O-GlcNAcylation status. Therefore, HG dysregulates O-GlcNAcylation and the related HIF1A pathway, thus preventing metformin-stimulated inhibition of growth and proliferation of SKOV-3 cells. The summary of our findings is presented in Figure 7.

## 4. Material and Methods

### 4.1. Reagents

Metformin, trypsin-EDTA, and Trizol^®^ Reagent were supplied by Sigma (St. Louis, MO, USA). Glucose free RPMI 1640 and fetal bovine serum (FBS) were supplied by Cambrex (Basel, Switzerland) and Life Technologies (Carlsbad, NM, USA). High Capacity cDNA Reverse Transcription Kit, TaqMan^®^ Gene Expression Assays, and TaqMan Universal PCR MasterMix were obtained from Life Technologies USA. All other chemicals and solvents were of high analytical grade and were supplied by Sigma or POCH S.A. (Gliwice, Poland).

### 4.2. Cell Culture and Treatment

The human SKOV-3 cell line used in the experiments was obtained from the American Type Culture Collection (ATCC) based in Rockville, MD, USA. The cells were grown in a monolayer at 37 °C in a 5% CO_2_ atmosphere in RPMI 1640 medium supplemented with 10% heat inactivated FBS, penicillin (10 U/mL) and streptomycin (50 μg/mL). In order to obtain normal glucose conditions (NG, 5 mM) and hyperglycemic conditions (HG, 25 mM), D-glucose was added to glucose-free RPMI 1640. Cells were cultured in growth medium with 5 mM glucose for 24 h before switching to 25 mM glucose. Then, the SKOV-3 cells were maintained for at least 1 day in NG or for 2 days in HG to achieve a logarithmic growth phase. The effect of metformin on the viability of SKOV-3 cells was determined by the MTT test (3-(4,5-dimethylthiazol-2-yl)-2,5 diphenyltetrazolium bromide) as described previously. We found that the ≥5 mM concentration of metformin evoked a significant decrease in the viability of SKOV-3 cells and the IC_50_ value was designated as 14 mM [13]. For further experiments, we chose a metformin concentration of 10 mM, which corresponded to a SKOV-3 survival rate of more than 60% of cells in relation to untreated cells. Finally, the cells were cultured under normo- and hyperglycemic conditions with or without metformin. The medium was changed every 24 h. The cells were routinely screened for Mycoplasma contamination.

### 4.3. Determination of Proliferation Rate 

To determine the SKOV-3 cell proliferation rate we employed the trypan blue exclusion method [37]. Live cells possess intact cell membranes that are impermeable to trypan blue and exclude the dye. After mixing the cell suspension with the trypan blue solution, a viable cell will have a clear cytoplasm whereas a nonviable cell will have a blue cytoplasm. The SKOV-3 cells were seeded at a density of 2 × 10^5^ per sample in growth media with 5 mM glucose for 24 h. Hyperglycemia was achieved by transferring cells from a 5 mM glucose medium to a medium containing 25 mM glucose. The adaptation time to the glycemic condition was 24 h. Then, the cells were cultured in NG for 24 h and HG for 48 h to reach the logarithmic growth phase. Then, metformin was added to obtain a final concentration of 10 mM. To calculate the proliferation rate, the cells were counted after 24, 48, and 72 h of exposure to metformin. Briefly, 4% trypan blue solution was mixed with the cell suspension in a ratio of 1:1, transferred to the chamber of Thoma and viable/nonviable cells were counted under an optical microscope. Based on the number of cells at the beginning and at each studied time point, we calculated the doubling time using the following formula td = t/log2 (Nt/N0), where td is time required for duplication of cell number, t is the time interval between the initial and final calculation of cell number, N0 and Nt are cell numbers at the beginning and the end of the experiment, respectively [38,39]. 

### 4.4. RNA Isolation and cDNA Synthesis 

Total RNA was isolated using Trizol^®^ Reagent (Sigma-Aldrich, St. Louis, MO, USA) according to the manufacturer’s protocol. An RNA ratio of 260/A280 ≈ 2.0 was considered as pure. Complementary DNA (cDNA) was synthesized using the High-Capacity cDNA Reverse Transcription Kit (Life Technologies, Grand Island, NY, USA) following the manufacturer’s instructions. cDNA synthesis was performed in a 20 μL volume that included 10 μL of total RNA (2 μg), 2 μL 10× RT Buffer, 0.8 μL of 25× dNTP Mix (100 mM), 2 μL 10× RT Random Primers, 1 μL of RNase Inhibitor, 1 μL of MultiScribe™ Reverse Transcriptase and 3.2 μL of nuclease-free water. The profile of time and temperature was as follows: 10 min at 25 °C, 120 min at 37 °C and 5 min at 85 °C.

### 4.5. Quantitative Real-Time RT-PCR (RT-qPCR)

Real-time gene expression analysis of target genes: OGT, OGA, SLC2A1 (GLUT1), SLC2A3 (GLUT3), HIF1A was performed using TaqMan^®^ Gene Expression Assays according to the manufacturer’s instructions. The HPRT1 (hypoxanthine phosphoribosyltransferase 1) gene was used as an internal control. The following assays were used to determine gene expression: OGT—Hs00269228_m1; OGA—Hs00201970_m1; SLC2A1-Hs00892681_m1; SLC2A3—Hs00359840_m1; HIF1A—Hs00153153_m1; HPRT1—Hs02800695_m1. PCR reactions were performed in a 10 µL volume that included 5 µL of 2× TaqMan Universal PCR MasterMix, 3.5 µL of water, 1 µL cDNA template (50 ng) and 0.5 µL of TaqMan^®^ Gene Expression Assay. The RT-qPCR reaction was carried out using the Mastercycler ep realplex. Relative RNA quantification was performed using the ΔCt method. Δ*C*t (*C*t_targeted gene_ − Ct_HPRT1_) values were recalculated into relative copy number values (number of target gene mRNA copies per 1000 copies of HPRT1 mRNA).

### 4.6. Morphological Assessment of Apoptosis and Necrosis

To determine the ratio between viable apoptotic and necrotic cell fractions, simultaneous cell staining with Hoechst 33258 and PI (propidium iodide) was conducted. These fluorescent dyes vary in their spectral characteristics and ability to penetrate cells. The analysis was performed with a fluorescence microscope (Olympus IX70, Tokyo, Japan). The morphological changes were detected at 24, 48, and 72 h of exposure to normoglycaemia and hyperglycemia with or without 10 mM metformin. At each time point studied, cells were removed from culture dishes by trypsinization, centrifuged and suspended in PBS to obtain 1 × 10^5^ cells/mL. To 100 µL of cell suspension, 1 µL of Hoechst 33258 (0.13 mM) and 1 µL of PI (0.23 mM) were added and the cells were incubated at room temperature for 10 min in the darkness [40]. At least 300 cells were counted on each slide and each experiment was done in triplicate. 

### 4.7. Statistical Analysis

Data were expressed as mean ± SD. Two-way analysis of variance was used. Statistical calculations were performed using STATISTICA ver.11. A *p*-value of <0.05 or <0.01 was considered as significant.
